# Weather temperature and the incidence of hospitalization for cardiovascular diseases in an aging society

**DOI:** 10.1038/s41598-021-90352-x

**Published:** 2021-05-25

**Authors:** Kihei Yoneyama, Michikazu Nakai, Takumi Higuma, Kanako Teramoto, Mika Watanabe, Toshiki Kaihara, Yoko Sumita, Yoshihiro Miyamoto, Satoshi Yasuda, Yuki Ishibashi, Masaki Izumo, Yasuhiro Tanabe, Tomoo Harada, Hisao Ogawa, Yoshihiro J. Akashi

**Affiliations:** 1 Division of Cardiology, Department of Internal Medicine, St. Marianna University School of Medicine, 2-16-1, Sugao, Miyamae-ku, Kawasaki, Kanagawa 216-8511 Japan; 2 Department of Statistics and Data Analysis, Center for Cerebral and Cardiovascular Disease Information, National Cerebral and Cardiovascular Center, Suita, Osaka Japan; 3 Department of Cardiovascular Medicine, National Cerebral and Cardiovascular Center, Suita, Osaka Japan

**Keywords:** Cardiology, Cardiovascular biology, Cardiovascular diseases

## Abstract

Weather temperatures affect the incidence of cardiovascular diseases (CVD), but there is limited information on whether CVD hospitalizations are affected by changes in weather temperatures in a super-aging society. We aimed to examine the association of diurnal weather temperature changes with CVD hospitalizations. We included 1,067,171 consecutive patients who were admitted to acute-care hospitals in Japan between April 1, 2012 and March 31, 2015. The primary outcome was the number of CVD hospitalizations per day. The diurnal weather temperature range (DTR) was defined as the minimum weather temperature subtracted from the maximum weather temperature on the day before hospitalization. Multilevel mixed-effects linear regression models were used to estimate the association of DTR with cardiovascular hospitalizations after adjusting for weather, hospital, and patient demographics. An increased DTR was associated with a higher number of CVD hospitalizations (coefficient, 4.540 [4.310–4.765]/°C change, *p* < 0.001), with greater effects in those aged 75–89 (*p* < 0.001) and ≥ 90 years (*p* = 0.006) than among those aged ≤ 64 years; however, there were no sex-related differences (*p* = 0.166). Greater intraday weather temperature changes are associated with an increased number of CVD hospitalizations in the super-aging society of Japan, with a greater effect in older individuals.

## Introduction

The prevention of cardiovascular disease (CVD) involves lifestyle patterns that are incorporated as daily habits. According to guidelines for the prevention of CVD development, clinicians and a team-based care approach should guide individuals to establish healthy living, healthy eating, regular exercise, proper sleep guidance, avoidance of tobacco smoking, the management of hypertension and diabetes mellites, and use of aspirin and statin therapy^1^. Daily habits are greatly influenced by daily variations in the weather. For example, people stay indoors and refrain from walking during stormy days and jog outside on sunny days. In this way, the weather and our daily habits are closely related. Weather forecasting helps people avoid danger, by knowing the maximum and minimum temperatures and natural disasters.

Previous studies have provided important information on how extreme weather temperatures affect morbidity and mortality^2,3^. For example, lower weather temperatures have been reported to be a risk factor for incident CVD^4,5^ and cardiovascular death^2,3^. Weather temperature can be used to guide the prevention of CVD.

However, there are three main limitations of previous studies with regard to discussion on the prevention of CVD. First, previous reports have typically selected specific heart diseases or relatively small study samples^2,3^. For example, the association of temperature with myocardial infarction, heart failure, or aortic disease is interesting; however, there is a need to prevent all CVDs in the general population. Second, although studies on the absolute effects of weather (hot, cold, or mean temperature) on CVD are well known^2–5^, few studies have focused on diurnal temperature changes. Therefore, clinicians are unsure on how to guide their patients to be careful of weather-induced temperature changes. Third, although Japan has the world's largest aging population, there are no studies on the relationship between weather-based temperature and CVD in a super-aging society^6^. In addition, there are no reports on the association between temperature and the development of CVD, based on an analysis of Japanese nationwide databases.

To address the issue, we conducted an observational study using a nationwide registry database, to investigate whether the number of CVD hospitalizations is related to weather temperature in the super-aging society in Japan. The Japanese Registry of All Cardiac and Vascular Diseases (JROAD) database includes all patients with CVDs who required hospitalization and constitutes a nationwide dataset in Japan. The study aims to assess the relationship between weather temperature and the development of CVD to guide clinicians on recommendations regarding the weather, to improve healthcare for an increasingly aging society.

## Methods

### Data collection

The JROAD database is a nationwide prospective registry that was designed to assess the clinical activity of each Japanese institution with regard to cardiovascular care and provide adequate feedback to teaching hospitals for improving patient care. A detailed description of the database design and methods has been published previously^7^. Briefly, the Japanese Circulation Society (JCS) developed the JROAD database, which includes demographic data from each hospital since 2004, and the JROAD-DPC nationwide database, which includes data from the Japanese Diagnosis Procedure Combination/Per Diem Payment System (DPC/PDPS) since 2014. The DPC database is a mixed-case classification system linked with a lump-sum payment system, which was launched in 2002 by the Ministry of Health, Labour and Welfare in Japan^8^. Compared with other registry databases, the Japanese DPC database enables researchers to conduct nationwide studies of both, descriptive and/or analytical epidemiology in a real-world clinical setting. The DPC database includes data on the following elements: demographics for each patient (e.g., age and sex); principal diagnoses (coded according to the International Classification of Diseases, 10th revision [ICD-10]); comorbidities at admission (ICD-10 coded); complications after admission (ICD-10 coded); procedures, including surgery, medications, and devices used during hospitalization; length of stay; discharge status; and medical expenses^7,8^. Institutions using the DPC system encompass a wide variety of centers, including academic, urban, and rural hospitals^7^. All data included in this study were from hospitalized patients with clinically apparent CVD.

We collated and used the dataset of the weather variables in Japan from the National Institute for Environmental Studies (http://www.nies.go.jp). The weather variables included hourly weather temperature and humidity. The weather variables were merged with the DPC database by using the acute-hospitalization day and municipal code provided by the Japanese Ministry of Internal Affairs and Communications (http://www.soumu.go.jp/denshijiti/code.html).

### Design

We conducted a cross-sectional study using data from the JROAD, JROAD-DPC, and weather variables between April 1, 2012 and March 31, 2015.

### Subjects

**Figure 1:**
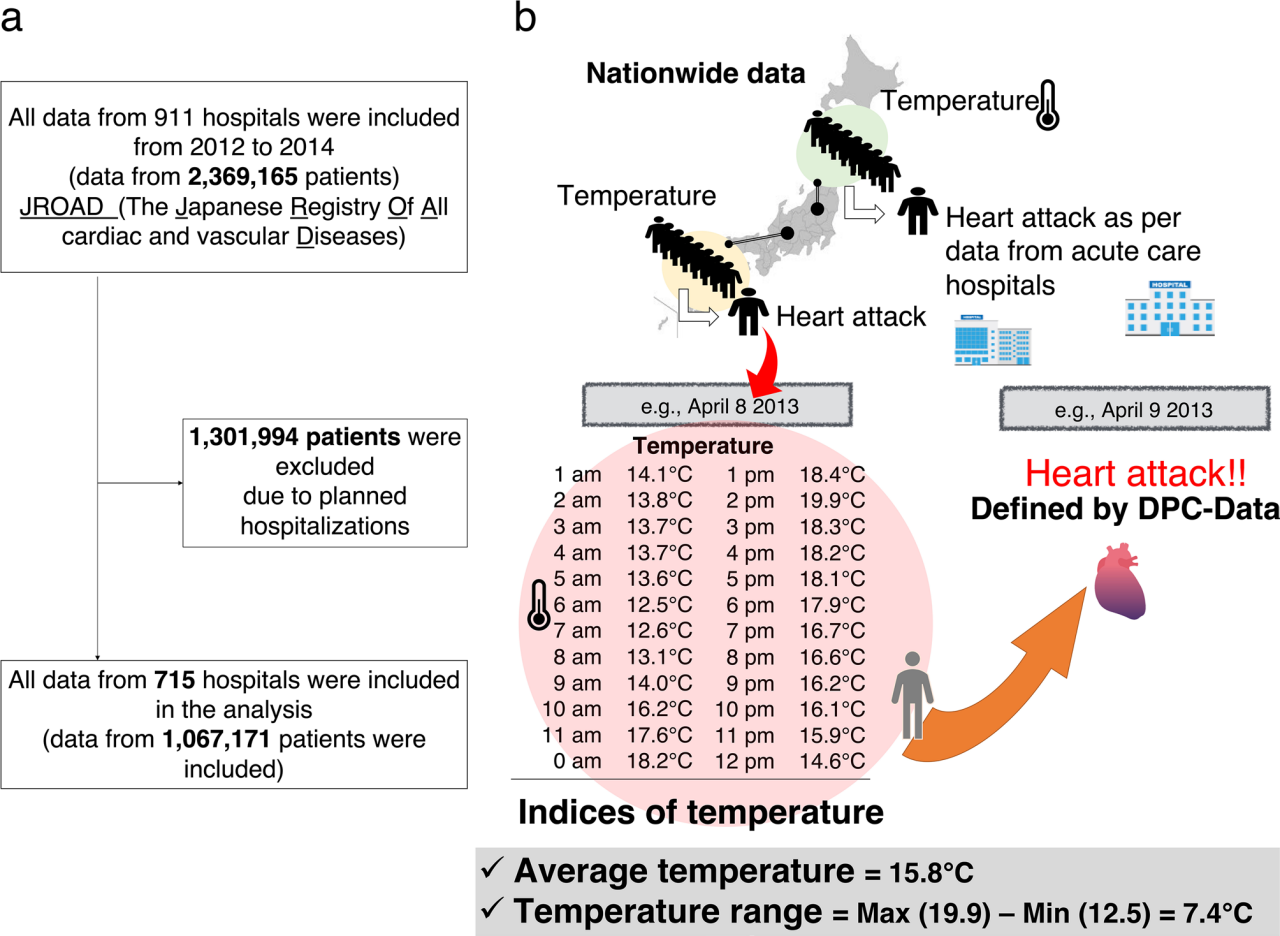
Flowchart of patient disposition. The flowchart presents the analyses conducted to evaluate an association between weather and incident cardiovascular disease.

In total, 911 hospitals were included in this study, and 2,369,165 consecutive patients were initially screened for study inclusion. However, 1,301,994 patients were excluded because of planned hospitalizations, which included admission for percutaneous coronary intervention for stable angina, follow-up catheterization, diagnostic admissions, scheduled catheter ablation (such as that for atrial fibrillation and supraventricular tachycardia), and scheduled cardiac surgery. In the final analysis dataset, we included data from 1,067,171 patients for statistical analysis (Fig. 1). This research was designed by the authors, and the study protocol was approved by the institutional ethics committee of the St. Marianna University of Medicine. Each hospital anonymized their patient IDs using code-change equations for the original JROAD-DPC data.

### Exposure

Seasons were separated into spring (March 23–June 21), summer (June 22–September 21), fall (September 22–December 21), and winter (December 22–March 22). The average temperature was defined as the average of each hourly temperature and humidity within a day. DTR was calculated by subtracting the minimum weather temperature from the maximum weather temperature in the same day. A higher index represented a greater change of weather temperature. The average and range of weather variables in a certain day were assigned to the day before an emergency hospitalization due to CVD.

### Outcomes

The primary outcome was the number of CVD hospitalizations per day. For the purposes of this study, CVD hospitalizations included coronary artery disease, heart failure, arrhythmia, aortic and peripheral artery diseases, cardiac arrest, pulmonary embolism, pulmonary hypertension, endocarditis/pericarditis, and congenital heart disease.

### Covariates

The average weather temperature and DTR as continuous and categorical values were subdivided into quintiles, that were adjusted for weather (season and average humidity), hospital (east/west Japan, number of hospital beds, and presence of coronary care unit, cardiac surgery service, and board-certified cardiologist), and patient demographics (age, sex, height, weight, smoking, Charlson Comorbidity Index, angina, acute myocardial infarction, heart failure, atrial fibrillation/flutter, aortic diseases, cardiac arrest, pulmonary embolism, pulmonary hypertension, and Tetralogy of Fallot).

### Statistical analysis

Descriptive statistics were analyzed for the demographics of hospital, weather, and patients. The number of cardiovascular hospitalizations by season was compared by one-way analysis of variance with Bonferroni post-hoc comparisons. We used multilevel mixed random-effects and population-averaged linear models to evaluate the association between the number of cardiovascular hospitalizations and weather variables. The multilevel mixed-effect models were used for evaluating the random effects of hospital variations (institutional code) determined by the JROAD study. The average weather temperature and DTR, as continuous and categorical values, respectively, were adjusted for weather, hospital, and patient demographics. The predicted number of cardiovascular hospitalizations per day was calculated using “marginsplot” after the creation of multilevel mixed random-effects and population-averaged linear models in STATA. The random effects and covariates were considered clinically important factors. All analyses were performed using STATA statistical software version 14 (Stata Corp, College Station, TX, USA). Statistical significance was defined as *p* < 0.05.

### Ethics approval

The study protocol was approved by the institutional ethics committee of the St. Marianna University of Medicine (#4038).

### Patient and public involvement

Patients and/or the public were not involved in the design, conduct, reporting, or dissemination plans of this research.

### Provenance and peer review

Not commissioned; externally peer reviewed.

## Results

Data were collected from a total of 1,067,171 consecutive patients with CVD admitted across 715 acute-care hospitals in Japan between 2012 and 2014. Patient characteristics and demographics are shown in Table 1.

**Table 1:** Patient characteristics.

	All
**Demographics (n = 1,067,171)**	
Age, year	71.3 ± 20.1
Female sex, n	451,159 (42.3%)
**Cardiovascular disease**	
Angina, n	110,348 (10.3%)
Acute myocardial infarction, n	112,738 (10.6%)
Unstable angina, n	57,041 (5.3%)
Atrial fibrillation or flutter, n	33,879 (3.2%)
Heart failure, n	31,4244 (29.4%)
Aortic disease, n	59,019 (5.5%)
Pulmonary embolism, n	12,718 (1.2%)
Cardiac arrest, n	91,657 (8.6%)
**Coexisting condition**	
Charlson Comorbidity Index score	1.7 ± 1.5
**Facility (n = 715)**	
No. of hospital beds, n	500 ± 239
No. of board-certified cardiologist, n	7.8 ± 8.6
**Weather**	
Average weather temperature, ℃	14.5 ± 8.7
Diurnal weather temperature range, ℃	7.7 ± 3.1

Values are presented as the mean ± standard deviation.

In this study population, the mean age was 71.3 ± 20.1 years and 42.3% of subjects were female. The incidence of acute myocardial infarction, heart failure, and cardiac arrest was 10.6%, 29.4%, and 8.6%, respectively. The overall hospital had a 500-bed count, and 59% of the study centers had cardiosurgical services. The mean weather temperature was 14.5 °C with intraday variations of up to 7.7 °C. Overall, the number of incident CVD hospitalizations tended to be higher in winter (Supplemental Fig. 1).

### Association between average weather temperature and CVD hospitalizations

Multilevel mixed-effects linear regression analysis indicated that a number of incident CVD hospitalizations were associated with lower average weather temperature (coefficient, − 3.962 [− 4.081 to − 3.3842] per ℃, *p* < 0.001) after adjustments for weather (season and average humidity), hospital (east/west Japan, number of hospital beds, presence of a coronary care unit, cardiosurgical service, board-certified cardiologist), and patient (age, sex, height, weight, Brinkmann Index, Charlson Comorbidity Index score, angina, acute myocardial infarction, heart failure, atrial fibrillation/flutter, aortic diseases, cardiac arrest, pulmonary embolism, pulmonary hypertension, and Tetralogy of Fallot) characteristics (Table 2).

**Table 2:** Association of weather temperature on the day of admission with hospitalization.

Hospitals, N = 1354	Multilevel mixed-effects linear regression; random effect; institution	
	Adjusted coefficient (95% confidence interval)	*p*-value
Average weather temperature (continuous values)	− 3.962 (− 4.081 to − 3.842)	< 0.001
**Average weather temperature (categorical values)**		
Q1 (< 5.7 °C)	Reference	
Q2 (5.8–11.3 °C)	1.139 (− 0.780 to 3.059)	0.245
Q3 (11.4–17.9 °C)	8.613 (6.367 to 10.86)	< 0.001
Q4 (18.0–23.3 °C)	− 27.966 (− 30.609 to − 25.324)	< 0.001
Q5 (> 23.4 °C)	− 82.74 (− 85.617 to − 79.864)	< 0.001
Weather temperature range (continuous values)	4.540 (4.314 to 4.765)	< 0.001
**Weather temperature range (categorical values)**		
Q1 (< 0.05 °C)	Reference	
Q2 (0.5–0.6 °C)	5.736 (3.833 to 7.639)	< 0.001
Q3 (0.7–8.2 °C)	9.695 (7.722 to 11.669)	< 0.001
Q4 (8.3–10.2 °C)	4.681 (2.649 to 6.714)	< 0.001
Q5 (> 10.3 °C)	39.282 (37.121 to 41.442)	< 0.001

Coefficients were adjusted for weather (season [spring, March–May; summer, Jun–Aug; autumn, Sep–Nov; winter, Dec–Feb] and average humidity), hospital (east/west Japan, number of hospital beds, and presence of coronary care unit, cardiac surgery service, and board-certified cardiologist), and patient (age, sex, height, weight, smoking, Charlson Comorbidity Index, angina, acute myocardial infarction, heart failure, atrial fibrillation/flutter, aortic diseases, cardiac arrest, pulmonary embolism, pulmonary hypertension, and Tetralogy of Fallot) characteristics.

**Figure 2:**
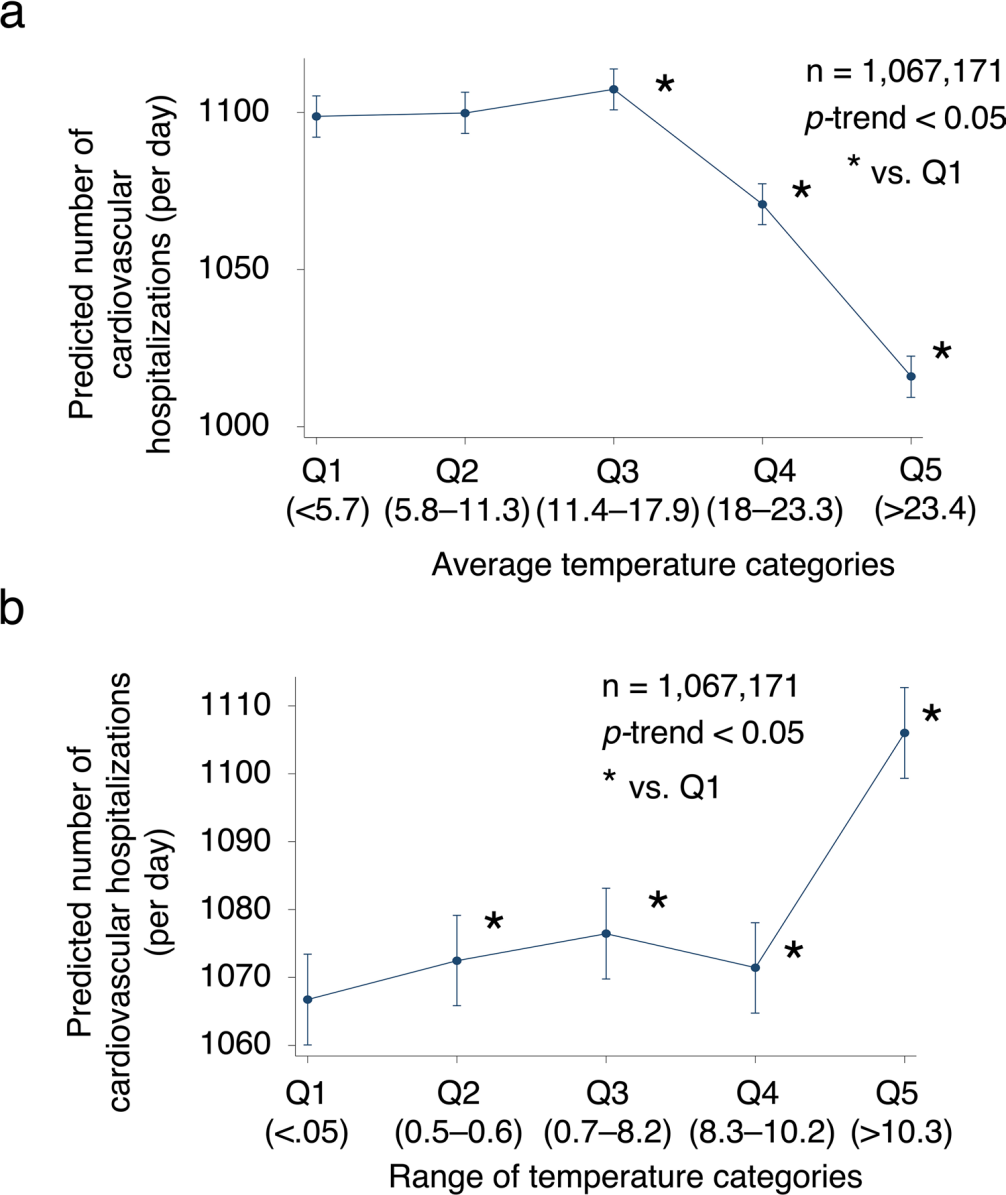
Association of weather temperature with acute hospitalizations for incident cardiovascular disease. The association of the mean adjusted probability of the number of hospitalizations due to cardiovascular disease across the quartiles of average weather temperature (**a**) and intraday weather temperature change (**b**). The bars indicate 95% confidence intervals.

The number of CVD hospitalizations was higher in the Q3 (coefficient, 8.613 [6.367–10.860] vs. Q1, *p* < 0.001), Q4 (coefficient, − 27.966 [− 30.609 to − 25.324] vs. Q1, *p* < 0.001), and Q5 (coefficient, − 83.74 [− 85.617 to − 79.864] vs. Q1, *p* < 0.001) groups of average weather temperature than in the Q1 group, after the above-described adjustments (Fig. 2a).

### Association between weather temperature changes and CVD hospitalizations

The increase in the diurnal weather temperature range (DTR) was associated with a greater number of CVD hospitalizations (coefficient, 4.540 [4.310 to 4.765] per ℃, *p* < 0.001) after adjusting for weather, hospital, and patient characteristics (Table 2).

The number of CVD hospitalizations was higher in the Q2 (coefficient, 5.736 [3.833 to 7.639] vs. Q1, *p* < 0.001), Q3 (coefficient, 9.695 [7.722 to 11.669] vs. Q1, *p* < 0.001), Q4 (coefficient, 4.681 [2.649 to 6.714] vs. Q1, *p* < 0.001), and Q5 (coefficient, 39.282 [37.121 to 41.442] vs. Q1, *p* < 0.001) groups of DTR than in the Q1 group, after the abovementioned adjustments (Fig. 2b).

### Effects of weather temperature indices in different age groups

**Figure 3:**
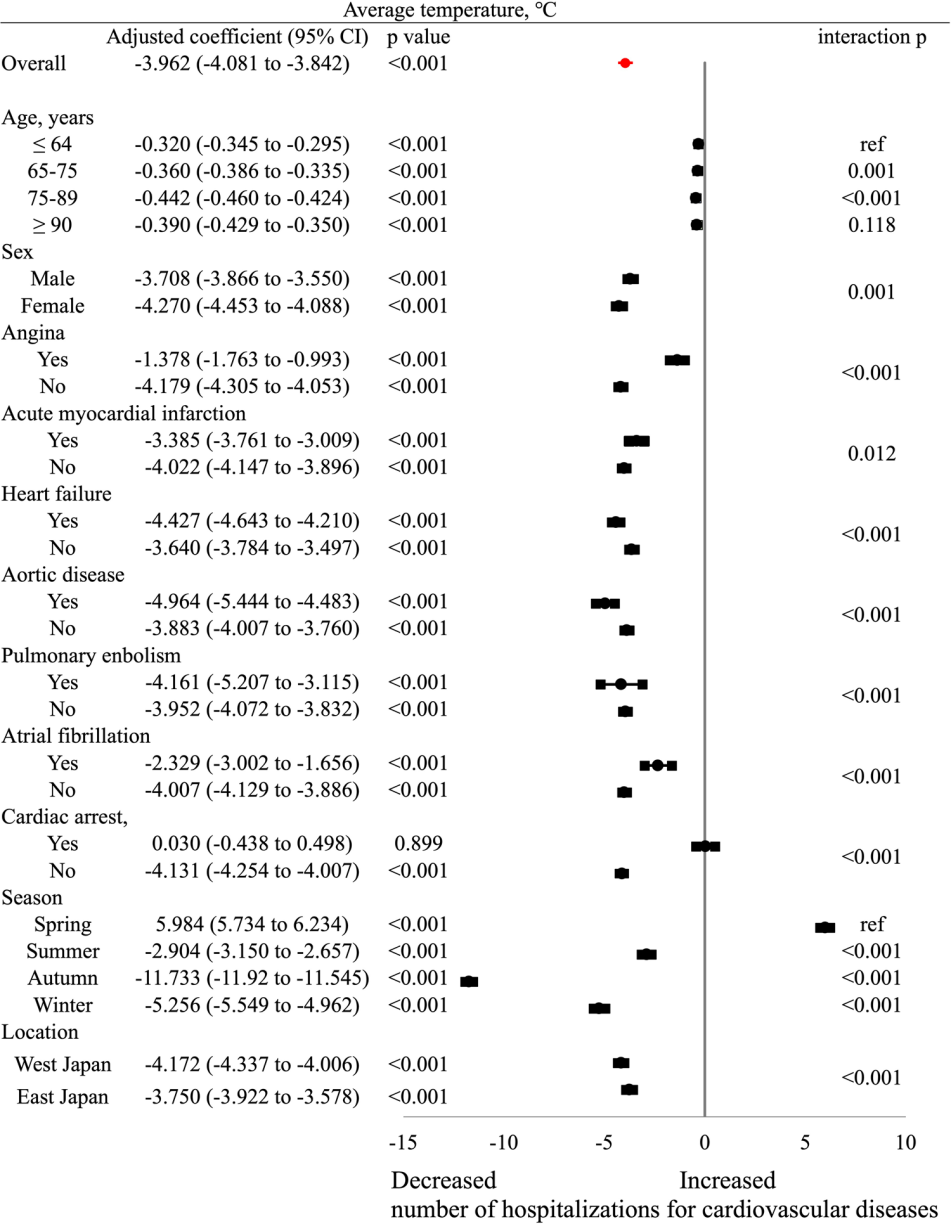
Association between average weather temperature and hospitalizations for cardiovascular conditions. Coefficients greater than zero represent an increase in the number of cardiovascular hospitalizations by the average weather temperature. The coefficient is indicated by a dot, and the lines represent the 95% confidence intervals (CIs).

The specific risk estimates of incident CVD with respect to average weather temperature are summarized in Fig. 3. A lower average weather temperature was associated with an increased incidence of CVD hospitalization for most patient conditions. Higher average weather temperatures were associated with an increased rate of hospitalization in spring. The effects of average weather temperature on all CVD admissions were greater in the 65–74 (coefficient, − 0.360 [− 0.386 to − 0.335], *p* < 0.001) and 75–89 (coefficient, − 0.442 [− 0.460 to − 0.424], *p* < 0.001) years age groups than in the ≤ 64 years age group; however, there were no differences between the ≥ 90 and ≤ 64 years age groups with regard to the effects of temperature on hospitalizations (*p* = 0.118). Moreover, the effects of average weather temperature were greater in female patients and individuals with heart failure, aortic disease, and pulmonary embolism than in patients without these risk factors (all *p* < 0.001).

**Figure 4:**
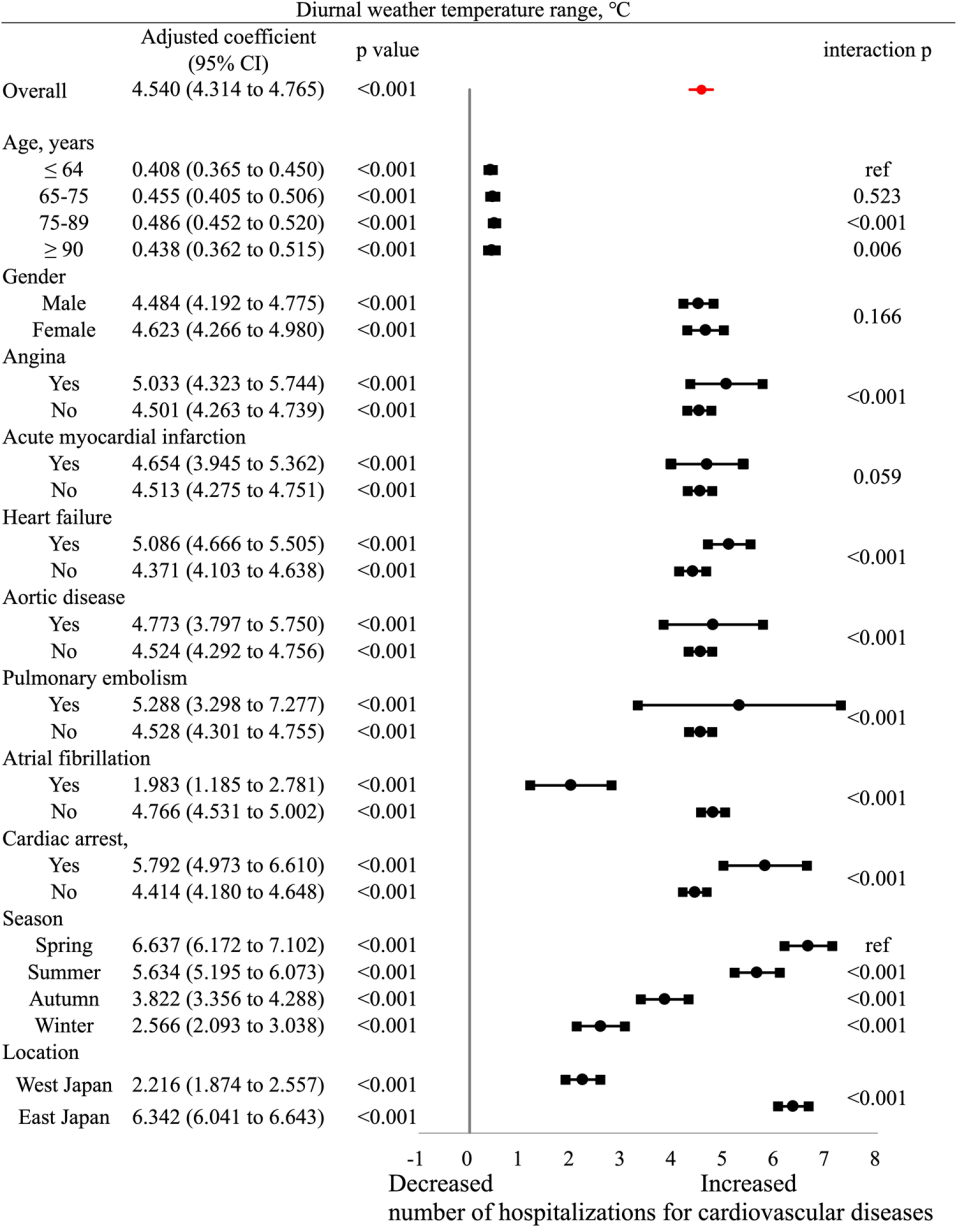
Association between weather temperature change and hospitalizations for cardiovascular conditions. Coefficients greater than zero represent an increased risk of incident cardiovascular hospitalization based on the range of weather temperatures. The coefficient is indicated by the dot, and the lines represent the 95% confidence interval (CIs).

A higher DTR was associated with an increased risk of incident CVD in all conditions (Fig. 4). The effects of an increased DTR on CVD hospitalization were greater in the 75–89 (coefficient, 0.486 [0.452–0.520], *p* < 0.001) and ≥ 90 (coefficient, 0.438 [0.362–0.515], *p* = 0.006) years age groups than in the ≤ 64 years age group; however, no sex-related differences were detected (*p* = 0.166). Greater effects of DTR were found in patients with angina, heart failure, aortic disease, pulmonary embolism, and cardiac arrest (*p* < 0.001 for all). The effect was greater in spring than in summer, autumn, and winter (*p* < 0.001 for all).

## Discussion

**Figure 5:**
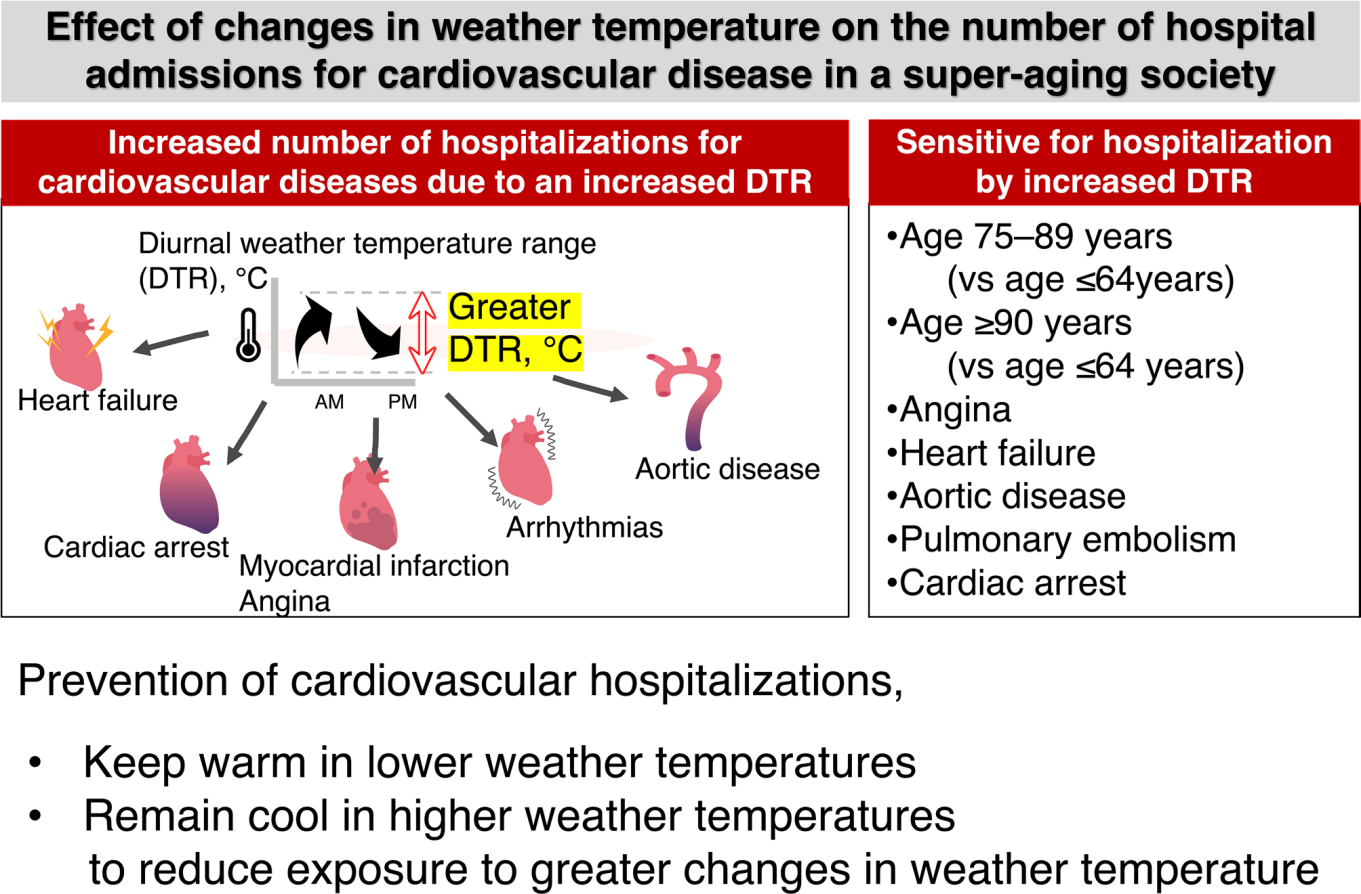
Illustration of the main study findings. A lower average weather temperature and a higher range of weather temperatures were associated with a higher incidence of cardiovascular hospitalization. These observations suggest that besides a reduction in weather temperature, a change in weather temperature may be considered for developing strategies for the prevention of cardiovascular diseases in an aging society.

We present a contemporary analysis of nationwide data from 1,067,171 patients and describe the estimated number of incident CVD hospitalizations. We found that overall, a higher number of incident CVD was associated with: (1) lower average weather temperatures and (2) greater weather temperature change, assessed by DTR. The effects of lower temperatures and DTR on all CVD admissions were more marked in elderly patients than in younger patients (Fig. 5). These observations suggest that in addition to average weather temperature, greater weather temperature changes need to be considered to evaluate the potential for the development of CVD in a super-aging society, such as Japan.

We found that lower weather temperatures were associated with increased hospitalizations for CVD. Other studies have found that cold-induced systemic hypertension^9^ and pulmonary hypertension^10^ are risk factors associated with the renin–angiotensin system, that could modulate the incidence of CVD hospitalizations. Using quantitative coronary angiography in 1980, Raizner et al. reported that the luminal diameter significantly decreased following 1 min of cold-pressor stimulation; this is known as coronary spasm^11^. Lower temperature ranges may be an additional environmental factor leading to higher incidences of hospitalization in cardiology wards.

However, Moghadamnia et al. conducted a systematic review and meta-analysis and concluded that both cold and hot weather temperatures increased the risk of cardiovascular mortality^12^. The mechanisms underlying higher weather temperature-related health effects potentially include impaired vascular endothelium, microthrombosis, elevated blood viscosity, disruptions in cholesterol levels, and dehydration^13,14^. The effects of high temperatures in our study were different; however, they may represent a change in the diurnal temperature and not an absolute effect of cold or hot weather.

Contrary to most previous studies, our study confirmed that a greater weather temperature range is an independent risk factor for CVD in a super-aging society. It has been reported that greater weather temperature range was significantly associated with emergent CVD admissions among the elderly in Beijing^15^ and South Korea^16^. A nationwide analysis reported that increased temperature variability was associated with a higher number of hospital admissions for CVD, ischemic heart disease, heart failure, heart rhythm disturbances, ischemic stroke, and arrhythmia^17,18^. The results from those previous studies are consistent with the findings of this study.

Compared with previous studies, however, we found that the effects of DTR on all CVD admissions increased with older age. The effects of DTR and cold temperature on CVD outcomes are not similar to those of other weather variables. The study by Vutkovici et al.^19^ found that diurnal variations in temperature are associated with a small increase in non-accidental mortality among the elderly population. Analitis et al. studied the short-term effects of cold weather on mortality in 15 European cities and demonstrated that the increased risk of CVD in cold weather was greater for older age groups^20^. In a review of recent studies, Astrom et al. explored the impact of heatwaves on morbidity and mortality in the elderly population^21^. These observations are consistent with the findings from this study. The elderly population is likely to develop complications due to atherosclerosis, infections, chronic obstructive pulmonary disease, cancer, dementia, Alzheimer’s disease, or low socioeconomic status^22,23^. The elderly population may have a lower thermoregulatory capacity and relatively weak immunological defenses, which could increase their vulnerability to respiratory tract infections in hot and cold weather^24^. Although there was no difference in the effects of average weather temperature on all CVD admissions between ages < 64 and > 90 years in this study, there were differences in the effects of DTR on all admissions. We believe that a change in temperature, rather than the absolute value of temperature, had a greater effect on the elderly.

We found that lower weather temperatures were associated with increased hospitalizations for CVD, particularly in winter. Data from a recent meta-analysis, that included 15 countries with a total sample size of 237,979 subjects, showed a seasonal pattern for most cardiovascular risk factors, with a higher prevalence in winter^25^. The potential pathogenic mechanism underlying these effects may be attributed to the effects of seasonal and weather temperature changes on CVD risk factors, such as increased blood pressure^26^, serum cholesterol^27^, platelets and fibrinogen activity^28^, endothelial dysfunction^29^, and respiratory infection in winter^30^. In this study, the effect of DTR was greater in spring than in summer, autumn, and winter. We could not identify the reason why a higher weather temperature in the spring conferred a higher risk of incident CVD in this observational study.

Furthermore, CVD hospitalization is a growing public health concern because of an aging population. Therefore, the findings of this study have important implications for risk reduction for CVD and may guide public health interventions to control and prevent the cardiovascular effects of exposure to changes in ambient and weather temperatures, particularly for individuals at high risk for hospitalization due to coronary attacks, arrhythmias, aortic disease, or heart failure. Keeping warm is important in the winter. Maintaining the main rooms at appropriate temperatures to avoid greater changes due to the weather will help prevent factors that can trigger an incident CVD.

One of the limitations of our study is that we included only Japanese Diagnosis Procedure Combination hospitals with cardiovascular beds that meet the JCS requirements. However, the JROAD is the largest cross-sectional study of nationwide cardiac health outcomes in Japan and constitutes a comprehensive database of epidemiological data for population-based studies. Cross-sectional studies do not determine cause and effect; however, the large sample size is a noteworthy strength of this study. Future longitudinal studies on the mechanism of temperature/DTR effects on cardiovascular events and their outcomes are needed. Determinants of the populations that are the most vulnerable to weather changes should be further explored. This study could not clearly identify whether the findings were related to the temperature itself or to the secondary effects on risk factors.

In conclusion, lower weather temperatures and greater intraday weather temperature variations are associated with an increased incidence of cardiovascular hospitalizations in the aging society of Japan. The effects of weather temperature change on all CVD hospitalizations were greater in older age. Keeping warm on cold days and cool on hot days may reduce the risk of CVD, especially in people aged ≥ 75 years. The results of this study may provide guidance to clinicians on instructing patients to remain cautious about weather-related temperature changes in a country with an increasingly aging society.

## Supplementary Information

Supplementary Information 1.

Supplementary Information 2.

## Data Availability

The data that support the findings of this study are available from the JROAD; however, restrictions apply to the availability of these data, which were used under approval for the current study and are thus not publicly available. Data are however available from the JROAD upon reasonable request. Environmental pollution data are available from the National Institute for Environmental Studies, Japan (http://www.nies.go.jp/db/index-e.html).
