# Supplementary material for: Weather temperature and the incidence of hospitalization for cardiovascular diseases in an aging society
Source: Sci Rep. 2021 May 25;11:10863. doi: 10.1038/s41598-021-90352-x (PMC8149862; doi:10.1038/s41598-021-90352-x)
Supplement: Supplementary file 2 — Supplementary Information 2. [file 41598_2021_90352_MOESM2_ESM.doc]

**SUPPLEMENTARY MATERIAL**

**Weather Temperature and the Incidence of Hospitalization for Cardiovascular Diseases in an Aging Society**

Kihei Yoneyama,^1^ Michikazu Nakai,^2^ Takumi Higuma,^1^ Kanako Teramoto,^1^ Mika Watanabe,^1^ Toshiki Kaihara,^1^ Kunihiro Nishimura,^2^ Yoko Sumita,^2^ Yoshihiro Miyamoto,^23^ Satoshi Yasuda,^2^ Yuki Ishibashi,^1^ Masaki Izumo,^1^ Yasuhiro Tanabe,^1^ Tomoo Harada,^1^ Hisao Ogawa,^2^ Yohishiro J. Akashi^1^

**List**

- Supplemental Table 1 - Association of weather temperature with cardiovascular hospitalizations
- Supplemental Figure 1 - Association between average weather temperature and the number of cardiovascular hospitalizations

| **Supplemental Table 1. Association of weather temperature with cardiovascular**  **hospitalizations** | | |
| --- | --- | --- |
| **A total of 1,354 hospitals** | **Multilevel mixed-effects linear regression;**  **Random effect; institution** | |
|  | **Adjusted coefficient (95% confidence interval)** | ***p*-value** |
| Average weather temperature (continuous values) | −3.962 (−4.081 to −3.842) | <0.001 |
| Average weather temperature (categorical values) |  |  |
| Q1 | Reference |  |
| Q2 | 1.139 (−0.780 to 3.059) | 0.245 |
| Q3 | 8.613 (6.367 to 10.860) | <0.001 |
| Q4 | −27.966 (−30.609 to −25.324) | <0.001 |
| Q5 | −-82.74 (−85.617 to −79.864) | <0.001 |
| Coefficients were adjusted for weather (season: Spring, March–May; Summer, Jun–Aug; Autumn, Sep–Nov; and Winter, Dec–Feb, and average humidity), hospital (east/west Japan, number of hospital beds, presence of coronary care unit, cardiac surgery service, board-certified cardiologist), and patient (age, sex, height, weight, smoking, Charlson Comorbidity Index, angina, acute myocardial infarction, heart failure, atrial fibrillation/flutter, aortic diseases, cardiac arrest, pulmonary embolism, pulmonary hypertension, and Tetralogy of Fallot) characteristics. | | |

**Supplemental Figure 1 - Number of incident cardiovascular diseases by season in Japan**

The number of incident cases of cardiovascular diseases tended to be higher in winter than in the other seasons.
